# Parturition and Neonatal Parameters of Three Species of Rhinoceros under Managed Care in the United States

**DOI:** 10.3390/ani13233653

**Published:** 2023-11-25

**Authors:** Emily E. Brenner, Lauren L. Howard, Jonnie Capiro, Jorge A. Hernandez

**Affiliations:** 1 White Oak, Yulee, FL 32097, USA; 2 San Diego Zoo Wildlife Alliance, Escondido, CA 92027, USA; drlaurenhoward@hotmail.com (L.L.H.);; 3 Department of Large Animal Clinical Sciences, College of Veterinary Medicine, University of Florida, Gainesville, FL 32610, USA

**Keywords:** rhinoceros, pregnancy, parturition, calf presentation, stillborn, dystocia, retained placenta, neonatal landmarks

## Abstract

**Simple Summary:**

Free-ranging rhinoceros populations are currently threatened due to habitat loss and poaching, and populations under managed care are not self-sustaining. Understanding theriogenology, the study of reproductive medicine, is important if these animals are to be managed effectively to create insurance populations against ecologically or anthropomorphically induced population declines. While there have been many studies evaluating the reproductive physiology and pathology of rhinoceros, there is still a lack of understanding regarding what is considered normal during birth and early calf development. By using video recordings and written medical records, we described the expected timelines for the phases of birth and calf development, as well as the frequency of anterior versus posterior calf positioning during birth and the prevalence of stillbirth. Equine parameters were used as a model for comparison for the results from this study due to the close taxonomic relationship between horses and rhinoceros. The results from this study will help guide clinical decisions for veterinary intervention during and after birth to achieve the best success for the health of the dam and the calf. Establishing references for birth and calf development will aid in sustaining populations of these endangered species in captivity, thereby creating a long-standing impact on the conservation of all rhinoceros species.

**Abstract:**

Rhinoceros species range from near threatened to critically endangered due to habitat loss and poaching. A sustainable ex situ breeding population is critically important to maintain genetic diversity and help ensure the survival of the species; however, not all populations under human care are self-sustaining. While rhinoceros reproductive physiology and pathology have been well studied, there is still a paucity of information describing the normal parameters of parturition and neonatal landmarks. Using video recordings, medical records, and keeper logs, we reviewed and compared data regarding the parturition of three rhinoceros species (black rhinoceros (BR) (*Diceros bicornis*), n = 4; greater one-horned rhinoceros (GOHR) (*Rhinoceros unicornis*), n = 21; and southern white rhinoceros (SWR) (*Ceratotherium simum simum*), n = 22) managed under human care in the United States. Using equine parameters as a model for comparison, we compiled the following data: the signs of impending parturition, durations of the parturition phases, calving presentation, frequency of dystocia or stillbirth, and time from birth to neonatal landmarks. Data from 47 births, including 26 videos, were examined. The durations of parturition phases I, II, and III had median lengths of 153 min (n = 18), 28 min (n = 21), and 205 min (n = 15), respectively. Anterior presentation of the calf was observed in 59% births, whereas posterior presentation occurred in 41% births. Posterior calving presentation was associated with a longer phase II of parturition (*p* = 0.04), although more data are needed to determine whether the posterior presentation of the calf carries a higher risk for stillbirth. Most (83%) stillbirths occurred in GOHR, indicating that this species might be at a higher risk for stillbirth compared to SWR (17%) (*p* = 0.07). The median time from birth to the calf standing was longer in the GOHR (64 min) compared to the SWR (30 min) (*p* = 0.02). Detailed descriptions of the parturition parameters and neonatal landmarks in rhinoceros will aid facilities with rhinoceros breeding programs to recognize abnormalities in the parturient or post-partum periods and guide indications for veterinary intervention.

## 1. Introduction

Free-ranging rhinoceros populations range from near threatened to critically endangered due to habitat loss and illegal trapping or hunting [1], and populations under managed care are not self-sustaining or have comparatively lower reproductive success [2]. As of 2017, only approximately half of southern white rhinoceros (*Ceratotherium simum simum*) females under managed care had reproduced successfully, with even lower success amongst rhinoceros that were born under human care versus those that were born in the wild [3]. Ex situ populations, defined as populations of rhinoceros outside their natural habitat, serve as important sources of insurance colonies against ecologically or anthropomorphically induced extinction [1]; however, they must have long-term viability to contribute to conservation. The lower reproductive success of managed animal populations can lead to decreased genetic diversity and population numbers [4]; therefore, understanding rhinoceros reproductive health is important if these animals are to be managed effectively under human care.

Reported causes for the decreased birth rates of rhinoceros managed under human care are primarily associated with factors that occur prior to birth. Examples include altered ovarian cycling and breeding behavior due to small herd compositions [3,5], the decreased fertility of females due to dietary phytoestrogens [6], and irregular ovarian cyclicity and acyclicity [2,7,8,9,10,11,12]. Additional examples include the increased development of reproductive pathology in females due to prolonged non-reproductive periods [13,14,15], decreased male reproductive fitness [16], and early embryo loss due to endometritis, pyometra, and luteal insufficiency [9,12,17,18,19]. Problems with late-term pregnancy and parturition, such as dystocia, abortion, and stillbirth, are rarely reported [9,19,20,21]; however, it is possible that these events are currently under-represented in the literature.

The current knowledge of rhinoceros theriogenology primarily involves the reproductive physiology and pathology [12,15,19,20,22,23,24,25,26,27], the diagnostic imaging of the reproductive tract [18,21,28,29,30,31,32,33,34], and breeding management. There are also multiple reports on the use of advanced reproductive techniques in rhinoceros, such as electroejaculation and sperm cryopreservation [35,36], artificial insemination [11,37,38,39], and sperm sex sorting [40,41]. However, few studies have evaluated rhinoceros during and after parturition [42,43,44,45,46,47]. This, in turn, makes it difficult to determine when veterinary intervention is advised in the peri-parturient and post-partum periods.

In this study, videos, medical records, and keeper logs regarding parturition events in the following species were evaluated: black rhinoceros (*Diceros bicornis*), greater one-horned or Indian rhinoceros (*Rhinoceros unicornis*), and southern white rhinoceros (*Ceratotherium simum simum*). All animals included in the study were managed under human care in the United States. The primary objective of this study was to establish a set of references regarding rhinoceros pregnancy, birth, and neonatal development. Equine parameters were used as a model for comparison due to the close taxonomic relationship between horses and rhinoceros. The secondary objective was to determine whether there is a significant correlation between the calving presentation and the risk for dystocia or stillbirth. The results from this project are intended to help guide clinical decisions regarding indications for veterinary intervention during the parturient and post-partum periods to achieve the best success for the health of the dam and the calf.

A recent study published by Dr. Hermes et al. in 2020 evaluated different birth parameters, gestational lengths, and serum progesterone concentrations in 19 southern white rhinoceros housed in Europe [42]. The findings in our study complement the work of Dr. Hermes by including additional species of rhinoceros and evaluating animals managed in the United States. To the best of the authors’ knowledge, this is the first study evaluating parturition in multiple rhinoceros species to determine a prospective standardization of the observational parturition parameters.

## 2. Materials and Methods

### 2.1. Animals

This study included rhinoceros from the following species: southern white rhinoceros (SWR), greater one-horned rhinoceros (GOHR), and black rhinoceros (BR). In total, documentation of 47 births consisting of videos (n = 26), written records (n = 35), or both were collected. These births occurred between the years 1995 and 2021. SWR (n = 22) and GOHR (n = 21) are equally distributed and well represented within this study, whereas only a small number of BR (n = 4) have been included thus far. All study animals are part of the ex situ population in the United States, representing 8 different zoological institutions.

Inclusion criteria for this study included female primiparous or multiparous rhinoceros of the species previously mentioned and their live or stillborn calves when applicable. Basic identifying information was collected on the sire when available. Javan rhinoceros (*Rhinoceros sondaicus*) and Sumatran rhinoceros (*Dicerorhinus sumatrensis*) were not included in this study, as neither of these species are currently housed in the United States.

### 2.2. Data Collection

This investigation was designed as an observational study. Data regarding the sire, dam, pregnancy, parturition, and neonatal landmarks were acquired using a data collection form and available video recordings of rhinoceros parturition that were submitted by participating institutions. Ethical approval and consent were granted from each individual institution by its research committee. Medical records, keeper logs, data collection forms, and video recordings were sent to a single observer either electronically or by mailing external hard drives to be remotely reviewed.

### 2.3. Video/Record Review

The quality, length, and equipment used for the video recordings varied widely and were not standardized for the purpose of this study. Videos were reviewed in their entirety to acquire as much information as possible, such as the durations of the parturition stages, position of the dam during parturition, fetal presentation during birth, and time from birth to significant neonatal landmarks. Not all videos were continuous, nor did they include all events; therefore, certain data points are not represented for every birth event. Some videos were supplemented with corresponding medical records or keeper logs. When video recordings were not available, information regarding parturition was collated from the animal’s medical records or keeper logs. Of the 47 birth events in the study, 26 events had videos included.

### 2.4. Definition of Labor Stages

Standard equine theriogenology definitions were used in this study for the calf presentation, position, and posture. In horses, anterior–longitudinal presentation, a dorsosacral position, and a posture of head and forelimbs extended are considered normal [48] (pp. 131–142). The phases of parturition were based off landmarks used in domestic equids, with some notable exceptions. In this study, the start of phase I of parturition was defined as an animal displaying two or more of the following signs: agitation, anorexia, tail curling or flagging, pressing of the hind end against the walls or bars of the enclosure, pawing, frequent urination or defecation, and repeatedly standing or lying down, or “restlessness”, and it ended with the presence of the amniotic sac at the vulvar lips. The start of phase II was defined by the presence of the fluid-filled amniotic sac at the vulvar opening, and it ended with the expulsion of the fetus. Phase III was defined as the time from the passage of the fetus to the expulsion of the fetal membranes.

### 2.5. Statistical Analysis

Data were calculated and reported as observed frequencies (n, %), medians, minimums, and maximums. In the analysis, 7 of 35 rhinoceros dams had multiple parturitions, for a total of 47 parturition events. Each parturition was considered an independent event. The observed frequencies of the calf presentation, calf position, calf posture, dystocia, assisted delivery, stillbirth, retained placenta, dam position during labor, and sex of the calf were compared between the GOHR and SWR by using a chi-square test. In addition, the gestation length (days), parturition phase I duration (minutes), phase II duration, and phase III duration, and calf time to sternal (minutes), nursing, and urination were compared between the GOHR and SWR by using the non-parametric Wilcoxon Rank Sum test. Black rhinoceros were not included in these comparisons due the small sample size. Finally, the durations of parturition phase I, phase II, and phase III (minutes) were compared between calves with anterior and posterior presentation by using the Wilcoxon Rank Sum test. Study animals with missing data were excluded from the selected statistical comparisons. In all analyses, values of *p* < 0.05 were considered significant.

## 3. Results

### 3.1. Signs of Impending Parturition

When available, certain late-gestation anatomical changes were evaluated, such as udder development, vulvar edema, and the leaking of milk. Udder development was first noted across the species at a median duration of 21 days prior to birth; however, in some cases, it was first seen up to 2 months prior to parturition (range: 2–60 days, n = 5). Vulvar edema and the loosening of the tail head and peri-vulvar muscles were noted at a median duration of 19 days prior to parturition (range: 10–26 days, n = 4). Milk leakage from the udder was first noted at a median duration of 1.5 days prior to birth; however, in one case, milk was reported leaking from the udder 11 days prior to parturition in a SWR (Table 1).

More immediate signs of impending parturition associated with phase I of labor were included in 19 birthing events in this study. Impending signs of labor in the rhinoceros included restlessness, vocalizing, agitation/aggression, horn pressing or rubbing, tail flagging or curling, hind-end rubbing or pressing, kicking out with the hindlimbs, frequent defecation or urination, pawing, anorexia, isolating behavior, and rolling or abdominal stretching. The most reported sign was restlessness (13/19, 68%), which is characterized by frequent changes of posture between standing and recumbency, as well as by the pacing of the enclosure. The next most reported signs were tail flagging/curling (8/19, 42%), followed by the rubbing or pressing of the hind end on the walls or bars of the enclosure (7/19, 37%). Aggressive or agitated behavior (6/19, 32%) and frequent urination or defecation (4/19, 21%) were also noted with some frequency (Table 2).

### 3.2. Gestation Lengths and Phases of Parturition

The gestation lengths ranged from 454 to 511 days across the species in our data. However, when comparing the gestation between species, SWR gestation was significantly longer (median: 504 days) compared to GOHR gestation (median: 482 days) (*p* < 0.01). The shortest gestation was associated with a BR (454 days); however, due to only one data point (n = 1), no significant comparisons could be made to the other species (Table 3).

Phase I had variable lengths in the rhinoceros ranging from 41 to 1770 min, with median times of 153 min across all species (n = 18), 145 min in the GOHR (n = 8), and 197 min in the SWR (n = 9). There was no statistical difference in the length of phase I between the SWR and GOHR. No comparison between species was made with BR, as only one data point was obtained; however, the duration of phase I in this rhinoceros was similar to what was seen in other species, with a length of 118 min (n = 1).

Phase II amongst the BR, GOHR, and SWR lasted between 5 and 200 min, with medians of 28 min across all species (n = 21), 63 min in the BR (n = 2), 31 min in the GOHR (n = 9), and 20 min in the SWR (n = 10) (Table 3). Most dams were in lateral recumbency during phase II of labor (80%, 21/26); however, occasional eutocic births occurred while the dams were standing (20%, 5/26) (Table 4).

Phase III amongst the BR, GOHR, and SWR lasted between 30 and 343 min, with medians of 205 min across all species (n = 15), 119 min in the GOHR (n = 6), 219 min in the SWR (n = 8), and one data point of 330 min for a BR. There was no statistical difference in the length of phase III between the SWR and GOHR (Table 3). Again, no comparison was made between the species with BR due to the low sample size. Retained placentas were noted with a prevalence of 12% (2/16) in our dataset (Table 4).

### 3.3. Calving Presentation and Sex of the Calf

The anterior presentation of the calf, where the head and forelimbs exit the birth canal first, occurred in 59% of all the rhinoceros births included in this study (17/29). When evaluated by species, anterior presentation was seen in 64% of the GOHR births (7/11) and in 60% of the SWR births (9/15). A total of 33% of the BR births were anterior presentation (1/3); however, this dataset includes only three births in total.

The dorsosacral position, in which the dorsal aspect of the calf faces the sacrum of the dam, was seen in 96% (25/26) of the births. The single delivery with abnormal positioning was a GOHR calf that was rotated laterally to a dorsosacral-iliac position while in posterior presentation. This position was associated with one of the two reported dystocias in our study, both of which occurred in GOHR dams (n = 2, 14% amongst GOHR, 6% amongst all births). All calves in the study displayed normal postures with the head, neck, and forelimbs or hindlimbs extended depending on anterior or posterior presentation (27/27, 100%). No births in this study required manual assistance during delivery. No statistical differences were noted in the calf presentation, position, or posture between the SWR and GOHR (Table 4).

Phase I of parturition tended to be longer in calves with a posterior presentation (n = 8; median = 219 min) compared to those with an anterior presentation (n = 9; median = 135 min), but the difference was not statistically significant (*p* = 0.37). However, phase II of parturition was significantly longer in calves with a posterior presentation (n = 8; median = 39 min) compared to those with an anterior presentation (n = 11; median = 21 min) (*p* = 0.04). Among the GOHR births specifically, phase II was notably longer in calves with a posterior presentation (n = 3; median = 80 min) compared to those with an anterior presentation (n = 6; median = 23 min) (*p* = 0.04). Phase III of parturition tended to be longer in calves with a posterior presentation (n = 4; median = 240 min) compared to those with an anterior presentation (n = 7; median = 175 min), but the difference was not statistically significant (*p* = 0.31) (Table 5).

Male calves occurred in 63% of births where the sex of the calf was known across all species (n = 32). Male calves also occurred more commonly within the species (100% in BR, n = 2; 59% in GOHR, n = 17; 62% in SWR, n = 13) (Table 4).

### 3.4. Live vs. Stillborn

Stillbirths were seen in 13% (6/46) of births where the calf viability was known. When comparing between species, the GOHR had more stillbirths (83%, 5/6) compared to the SWR (17%, 1/6), although the difference was not statistically significant (*p* = 0.07). Amongst the GOHR births, 24% (5/21) of the births were stillborn, whereas only 5% (1/22) of the SWR births were stillborn. No stillbirths from BR were included in this study, likely due to the small sample size (Table 4).

Two of the five stillbirths (40%) amongst the GOHR births were associated with posterior presentation. Both of these stillbirths occurred with the same dam. The first posteriorly presented stillbirth was associated with dystocia, whereas the second stillbirth occurred from an uncomplicated delivery. The calf presentation amongst the other three GOHR stillbirths and the one stillborn event in the SWR was unknown. The clinical significance of the posterior presentation of the calf and increased risk of stillbirth are currently difficult to determine due to the small sample. The data suggest an over-representation of GOHR amongst the stillbirths; however, more data are needed to validate this finding.

### 3.5. Dystocia

Dystocia was seen in 6% (2/35) of births where parturition was observed (Table 4). Two of the five stillbirths (40%) amongst the GOHR births were associated with dystocia. The first dystocia was associated with fetal malposition (posterior presentation with dorsosacral-iliac position). The calf presentation of the other stillborn dystocia was unknown.

### 3.6. Neonatal Landmarks

Calves achieved sternal recumbency between 1 and 105 min after birth, with a median time of 7 min (n = 18). Time to standing occurred between 20 and 195 min after birth, with a median time of 52 min (n = 19). When comparing the GOHR and SWR calves, the calf times to sternal and standing were shorter in the SWR calves compared to the GOHR calves (*p* = 0.04, *p* = 0.02). Calves were first noted to be successfully nursing between 65 and 450 min, with a median time of 127 min (n = 15). No statistical difference was noted when comparing the time to nursing between species. The time of first urination was only documented in two calves, one GOHR and one SWR, which occurred 193 and 389 min after birth, respectively. The passage of meconium was described in the medical records from a total of four animals. This occurred anywhere from 26.5 h to 72 h after birth (Table 6).

One GOHR calf included in the study had delayed neonatal development and was considered an outlier in our dataset. The longest durations of the times to sternal (105 min) and standing (195 min) in this study were associated with this calf. The birth occurred inside in a boma bedded with straw and the calf presented in posterior presentation, but the birth was considered uncomplicated. This calf was born with a valgus malformation of the right hind foot that later self-resolved.

## 4. Discussion

### 4.1. Introduction Statement

This is the first study in the United States to evaluate and report rhinoceros parturition events as well neonatal landmarks from data acquired through a video recording database. Additionally, this is the first report of its kind to include GOHR and BR. It is important to note that the submission of the rhino parturition information and videos was voluntary; therefore, our dataset is not comprehensive. Our primary focus of analysis was aimed towards describing the signs of impending parturition, determining the lengths of the three phases of parturition, identifying normal fetal presentations during birth, and establishing an expected timeline of neonatal development, such as the time to standing and nursing. These established guidelines will help rhinoceros breeding facilities and veterinarians to know what to expect and, consequently, when to intervene.

### 4.2. Signs of Impending Parturition

Late-gestation anatomical changes, such as udder development and vulvar edema, are useful tools to give caretakers an indication of impending parturition. This can be especially helpful when the date of breeding is unknown and, therefore, the expected due date for the dam falls within a large window of time. The timing of these changes is also important to ensure the continued health of the pregnancy, as early udder development or vulvar swelling in horses can indicate problems, such as placentitis, and, in some cases, are followed by abortion [49]. Additionally, early milk leakage from the udder can lead to the loss of the colostrum prior to the birth of the neonate, resulting in the failure of passive transfer. This may necessitate further assistance from veterinary professionals and zoological caretakers to provide alternative oral colostrum or parenteral immunoglobulins in addition to close monitoring for evidence of perinatal infection or illness due to poor passive immunity. Our findings can assist caretakers of gravid rhinoceros by providing guidelines for the expectation of anatomical changes associated with late gestation and indications for when intervention may be warranted due to possible upcoming adverse events, such as abortion.

In our study, there was a fairly large range of time for when udder development was first noted prior to birth. This range could be due to differences in the close visual access to the rhinoceros, the intensity of the monitoring or recording of anatomical changes in pregnant dams, as well as the level of experience or knowledge of the observers regarding changes in the udder. A median of 21 days, however, correlates with findings in other reports. In Hermes’s study, it was found that the udder development in SWR was typically noted three weeks prior to birth [42], and a review of rhinoceros behavior from 2006 reported udder development in multiple species around 30 days before parturition [43]. The literature regarding GOHR describes an earlier udder development of 6–12 weeks prior to birth [44,50]. Our dataset is too small (n = 5) to compare differences between species, although the two earliest data points of udder development (60 days and 25 days prior to birth) were associated with GOHR. Our findings, as well as other reports of udder development, mimic the timeline of udder development in horses, with the expected development anywhere between 0 and 4 weeks prepartum [48] (pp. 114–130).

Vulvar edema and the loosening of the tail head and peri-vulvar muscles that occurred at a median of 19 days prepartum in our study are similar to what was reported in Hermes’s study of SWR, in which genital swelling was typically noted 2 weeks prior to birth [42]. Reports in other literature of vulvar edema are less frequently discussed than udder development, with one review stating an average of 30 days before parturition [43].

Milk dripping, or “teat waxing”, is an important predictor of impending parturition in horses, as it should occur within 1–4 days before the beginning of phase I of labor [48] (pp. 114–130). In our study, we found that reports of milk dripping from the teats occurred within 0–48 h prior to birth, apart from one SWR dam that started to leak milk 11 days earlier. This pregnancy was otherwise normal, followed by a eutocic birth and a live calf.

### 4.3. Gestation Lengths and Phases of Parturition

The gestation lengths across the three species of rhinoceros included in this study lasted, on average, about 15 months, with a range of up to 57 days. This large range, however, is due to significant differences in the gestational length when compared between species, with SWR gestation lasting longer than GOHR gestation. This finding is consistent with those published in the literature [28,42,45].

The definitions of the phases of parturition and the determination of their lengths are important endeavors in veterinary medicine to allow for the recognition of peripartum disorders and the initiation of intervention when necessary. Our study evaluated parturition primarily through video recordings of rhinoceros housed in isolated enclosures in preparation for birth. Some video recordings only included one or two of the three phases of parturition or were not continuous recordings; therefore, data points for all phases of parturition were not collected for every birth event. In these cases, data points were supplemented with medical records or keeper logs when possible.

Phase I of labor is typically associated with the repositioning of the fetus in preparation for birth, initial uncoordinated uterine contractions, and cervical dilation. In domestic horses, the start of this phase is often easily recognizable and typical signs include flank watching, restlessness, rolling, anorexia, and sweating. The start of phase I was not as clearly visible in the rhinoceros in our study. To create a repeatable and reliable start time for phase I, we defined the start of phase I by the display of two or more of the signs reported in Table 2. Restlessness was the most common behavior noted in our study (68%). This finding is reflected in the literature, as it is the one behavioral change that is noted across all publications [42,43,44,46]. The second most common behavior in our study was tail curling and rubbing or the pressing of the hind end against the edges of the enclosure (42% and 37%, respectively); however, these behaviors are not described in other reports. Aggressive behavior and frequent urination or defecation were reported somewhat commonly in our study (32% and 21%, respectively) and are frequently described in the literature [42,43,44,46]. We believe that our definition of phase I is useful and repeatable, as it allowed for the recognition of phase I of labor in all videos where sufficient recordings were provided. Additionally, it is important to note that while restlessness is the most frequently observed behavior, incorporating other signs of impending labor, such as increased aggression, frequent urination or defecation, and the rubbing of the hind end, will help to provide a more accurate and earlier recognition of the start of labor in rhinoceros.

The length of phase I in our study was highly variable, ranging from as short as 41 min up to 29.5 h. However, this range includes two individuals with significantly longer phase I durations. These individuals were a SWR and a GOHR with phase I lasting 24 h and 29.5 h, respectively. Both births were considered eutocic with the delivery of live calves; however, the calf presentation in both was posterior. The longest reported phase I in this study was associated with a calf with a congenital valgus deformity of the right hindlimb. With the exclusion of these two outliers, the duration of phase I ranged from 41 min to just shy of 6 h (355 min). This complements both Hermes’s findings, in which the first stage of labor started approximately 5–7 h prior to birth [42], and findings in equine medicine, with phase I typically lasting about 1–4 h [48] (pp. 131–142). Other reports of rhinoceros parturition are vague when referencing the duration of phase I of parturition and do not provide any definition for the start of this phase other than to describe a variety of behavior changes seen up to 24–48 h prior to birth [43,44,46].

Phase II of parturition is characterized by active labor with concerted abdominal and uterine contractions ending with the expulsion of the fetus. In horses, phase II begins with the characteristic “water breaking”, which is caused by the rupture of the chorioallantoic membrane and the subsequent release of allantoic fluid as the fetus enters the birth canal. This is swiftly followed by active labor and the passage of the fetus. In rhinoceros, however, there is a prolonged lag time of up to two hours between the water breaking and the start of active labor. In our study, we found that the “water breaking” could be easily missed or difficult to differentiate from urination due to the variable quality of the videos. Alternatively, the first reliable visible sign of phase II was the presence of the fluid-filled amniotic sac at the vulvar opening. This appears as a shiny, round but taut, white tissue protruding from the vulva. Additionally, the time from when the allantoic sac is present at the vulvar opening until the time that the fetus is expelled is more characteristic of the phase II seen in horses, as it includes the portion of active uterine contractions. Therefore, the presence of the amniotic sac until the passage of the fetus was used as the definition of phase II in our study. Hermes’s 2020 study used a different approach by dividing phase II into two stages. The first stage incorporated the time between chorioallantoic rupture until the fetal membranes were visible at the vulvar opening, whereas the second stage incorporated the active labor portion [42].

The lengths of phase II in our study had median durations of 28 min across all species, 63 min in the BR, 31 min in the GOHR, and 20 min in the SWR. With the exception of the BR, these median values align with the expected duration of 20–30 min of phase II in horses [48] (pp. 131–142). The significance or validity of the longer duration of phase II in the BR noted in our study cannot be determined due to the small sample size (n = 2). Reports of the duration of phase II in the literature include one description of a Sumatran rhinoceros birth in which the phase II of labor lasted 2–3 h; however, this likely included the duration from the water breaking until the expulsion of the fetus, and therefore it is difficult to compare to our data [46]. A detailed description of a GOHR birth described a length of 28 min from the presence of the amniotic sac until the delivery of the calf [44], which fits with our findings. Additionally, Hermes’s study found that the active labor portion of phase II in SWR lasted between 12 and 16 min, which is also similar to our findings [42].

What is interesting to note is that longer durations of phase II can be associated with eutocic births and live healthy calves in rhinoceros. In horses, dystocia is defined as a prolonged duration of phase II of labor resulting from the cessation of the forward progress of the foal in the birth canal. The failure of the amniotic sac or fetal hooves to appear within 20 min of the chorioallantoic rupture is often cause for veterinary intervention, with a 10% increased risk of foal mortality for every 10 min of increased duration of phase II [51]. Our data suggest that rhinoceros calves can tolerate a much longer phase II with no apparent health effects, even when active labor lasts up to 10 times as long as horses. Additionally, phase II in horses almost always occurs with the mare in lateral recumbency [52]; however, the rhinoceros in our videos delivered calves in both recumbent and standing positions. Births occurring while standing have also been described in wild-born rhinoceros [43].

Phase III is characterized by the passing of the placenta and uterine involution and is defined as the time from the passage of the fetus to the expulsion of the fetal membranes. The length of phase III in our study was consistent across species and with other reports of rhinoceros births. In our study, phase III lasted a median duration of 205 min (3.4 h). Hermes’s study reports the placenta passing around 3 h after birth [42], and one report of a GOHR birth describes phase III lasting 3 h and 10 min [45]. Horses are expected to pass fetal membranes within 3–4 h after birth; otherwise, the placenta is considered retained, which can lead to possible life-threatening complications, such as metritis, endotoxemia, and laminitis [48] (pp. 131–142). While the rhinoceros in our study typically passed the fetal tissues under four hours, there is evidence that rhinoceros can tolerate phase III of parturition up to 6 h without any apparent medical concern.

Reports of retained placentas amongst rhinoceros are scarce, with only two occurrences in our dataset, leading to a prevalence of 12% (2/16). The reported prevalence of the retention of fetal membranes in horses can vary by species but typically falls between 2 and 17% and is often associated with a prolonged phase II of birth [48] (pp. 131–142) [53]. One SWR rhinoceros in our dataset did not deliver the placenta for over two days, at which point the dam was administered oxytocin and was able to pass the remaining placental tissue. This event was associated with an uncomplicated birth, producing a live calf that was delivered in anterior presentation; however, the calf was hand-reared due to low maternal care in the first 48 h. The second account of a retained placenta occurred in a primiparous GOHR with the delivery of a stillborn calf in posterior presentation and a prolonged phase II (50 min). There is no information on the exact length of phase III for this delivery, but medical records indicate that the placenta was retained. Additionally, no information was available to indicate whether this animal received any treatment for the retained placenta. Placental tissue was submitted for histopathologic evaluation, which showed adenomatous hyperplasia, neutrophilic placentitis, and villous fibrosis. The neutrophilic inflammation was suspected to be a result of the delayed passage. This dam had delivered a stillborn calf two years prior associated with dystocia and a prolonged phase II of labor (80 min) due to fetal malpositioning (posterior presentation, dorsosacral-iliac position). Unfortunately, there is no information regarding phase III from this stillbirth and whether the placenta was retained at that time as well. Currently, more data are needed to substantiate the trend that a prolonged phase II of labor carries a higher risk of the retention of placental tissues similar to what is seen in horses [48] (pp. 131–142).

### 4.4. Calving Presentation and Sex of the Calf

Determining the normal fetal presentation, position, and posture for rhinoceros is important, as abnormalities in these categories can lead to dystocia and may require veterinary intervention if the viability of the calf, and in some cases the dam, are to be maintained. However, there is a paucity of current literature regarding this subject. Our data included 29 births where the presentation of the calf was known, with an approximate 60/40 ratio of anterior-to-posterior calf presentation. These data suggest that both anterior and posterior presentation occur frequently in rhinoceros. When evaluated by species, no significant difference exists in the prevalence of posterior birth. The percentage of posterior presentation is lower in Hermes’s study of SWR, in which 15.7% (3/19) of births were associated with posterior presentation [42]. Only 1 out of 26 (4%) calves in our study presented with an abnormal dorsosacral-iliac position; the remainder of the births involved the normal dorsosacral positioning of the calf. No instances of transverse presentation, dorsopubic positioning, or abnormal calf posture with the flexion of limbs or neck occurred in our data, nor are they described in Hermes’s study [42].

These findings are significantly different from what is found in horses, in which anterior presentation is considered normal and abnormal fetal postures and positions are widely documented [48] (pp. 131–142). It is important to note, however, that the posterior presentation of calves in rhinoceros tends to be associated with a longer phase II of parturition, particularly in GOHR, in which the median time of phase II reaches 80 min in posterior presentation as opposed to 22 min in anterior presentation. Births in which calves presented posteriorly also led to longer phase II times in Hermes’s study, with one case of active labor lasting up to 47 min [42]. This is concerning, as a prolonged phase II of parturition can lead to the periparturient asphyxia of the fetus if the umbilical cord is compressed while passing through the birth canal. Two out of twelve (17%) posteriorly presented calves in our study were stillborn, whereas all the calves born in anterior presentation were viable. Two out of six stillborn calves (33%) were associated with posterior presentation; however, the calf presentation is unknown in the remaining four stillbirths in our study. Similarly, in Hermes’s study, the one stillbirth that occurred was associated with a posterior presentation, whereas all the anterior-presentation births resulted in live calves [42]. These findings suggest that posterior presentation, while commonly seen, could carry a higher risk for calf viability due to prolonged labor, especially in GOHR. More stillbirth events are needed, however, to determine whether the posterior presentation of the calf carries a higher risk for stillbirth. The cause for these species differences is currently unknown and further research is needed to determine whether this is due to anatomic or genetic differences, as is seen with different breeds or categories of horses [52].

It is also interesting to note that there was a skewed sex ratio with a higher percentage of male calves (63%) amongst all the species of rhinoceros in our data. While this skew may be due to the overall small sample size of calves where sex was known (n = 32), this finding could have significant impacts on the conservation efforts for the ex situ rhinoceros population by creating limitations for the resources to house solitary surplus males. Sex skews have been previously identified in the BR population in the United States [54].

### 4.5. Live vs. Stillborn

Our findings show that 13% (6/46) of all the calves delivered were stillborn. This rate is notably higher than what has been reported for horses, with a prevalence between 1.2 and 4.5%, depending on the report [51,55]. The discrepancy is due to both the decreased management and monitoring of rhinoceros dams undergoing parturition as well as the difficulty of implementing veterinary intervention safely during parturition when compared to domestic animals. The majority of stillbirths in our study (83%) occurred in GOHR dams, and about one-quarter of all GOHR births resulted in stillborn calves. This strongly suggests that GOHR are at an increased risk for stillbirth. This finding is corroborated by other reports [9], including a retrospective analysis of captive GOHR births from 1971 to 2010 in which 24% of births resulted in stillborn calves or perinatal death [47]. The cause of the stillbirth in many instances is unknown, and not all stillborn calves are associated with dystocia [45], but the retrospective study suggested that some stillbirths may result from unrecognized dystocia, as the signs of labor can be difficult to recognize in rhinoceros. One stillbirth occurred in the Hermes study, resulting in a prevalence of 5.3% (1/19) amongst their SWR cohort. This matches our prevalence of 5% (1/22) of stillborn calves amongst the SWR births in our data. The presentation of the stillborn SWR calf in our study is unknown; however, the calf presented posteriorly in the stillbirth reported in Hermes’s study, in addition to a stillbirth described in an SWR in Europe [42,45]. A report of the reproductive performances of SWR under managed care in South Africa noted a stillbirth prevalence of only 1.2% (7/562). However, the authors state that this represents a minimum estimate of stillbirths, as these animals were managed on an extensive property with predators present; therefore, there were likely additional stillbirths or abortions that were not documented [3]. No stillbirths were noted amongst the BR in our study, but this was likely skewed by the small sample size (n = 4). The current literature reports only one stillbirth in a BR [19], with one additional report describing a late-gestation abortion in a multiparous BR [20].

### 4.6. Dystocia

According to our data, dystocia appears to be rare in rhinoceros, with a prevalence of only 6% (2/35). This is low compared to the prevalence of 10.1% in horses according to a retrospective study from 2012 [51]. This retrospective study found that dystocia in horses is most commonly due to fetal postural abnormalities (96.8%), with feto-pelvic disproportion (i.e., hip lock), premature placental separation (i.e., red bag), and periparturient uterine hemorrhage only accounting for 3.2% of the dystocias combined.

Only two dystocias were included in our study; both were associated with GOHR, resulting in a 14% prevalence of dystocia amongst the GOHR births (2/14). One dystocia involved a nulliparous dam and was associated with fetal malpositioning, resulting in a stillborn calf due to a prolonged phase II. The second dystocia involved a multiparous dam and resulted in a stillborn calf; however, there is no additional information to indicate the cause for the complicated birth. Only one report in the literature describes an abnormal birth and veterinary intervention in rhinoceros. This report involves a multiparous 17-year-old GOHR under managed care with a prolonged phase II time lasting 31 h before a fetotomy was performed. The dystocia was caused by the fetal malpositioning of the calf due to a flexed forelimb. Post-mortem evaluations revealed that this calf had multiple congenital deformities, including cerebral aplasia. The dam recovered and resumed ovarian cycling after the fetotomy; however, the report does not describe whether the dam produced any live calves following this procedure [47].

More data are needed to determine the common causes of dystocia in rhinoceros, although, based on the few reports available, fetal positioning appears to be an important factor. To the best of the authors’ knowledge, there are no reports of premature placental separation or periparturient uterine hemorrhage in rhinoceros; however, the development of these pathologies is possible. It is likely that dystocia in rhinoceros occurs more commonly than what is reflected in the literature; however, complicated births still appear to be rare when compared to equids. This is presumably a result of the compact build of rhinoceros calves, where the lack of long slender legs and necks makes abnormalities of fetal posture less likely.

### 4.7. Neonatal Landmarks

Reports of neonatal landmarks after birth in rhinoceros, such as the time to standing or nursing, are sparse. However, knowing what is expected after a calf is born is important to be able to recognize when certain milestones are delayed and could indicate possible abnormalities. Nineteen calves were included in this study, although not all landmarks were included for each calf. Based on a video review, if the birthing stall was well bedded with absorptive or non-particulate substrate, such as hay or straw, a healthy calf would pull itself into sternal recumbency within 10 min, with a median duration of 7 min. Calves were standing by around 1 h after birth, with a median time of 52 min, which correlates with Hermes’s finding of calves standing about 55 min after birth [42].

Interestingly, we found that the SWR calves stood faster than the GOHR calves, with median times of 30 min amongst the SWR and 64 min amongst the GOHR. The cause for this is unknown and could be associated with external factors, such as the substrate used in the different housing styles for these two species, as the SWR were more commonly housed in large stalls, whereas many GOHR gave birth in smaller boma-type enclosures. More data are needed to determine whether this is a consistent finding between the two species regardless of the substrate or birthing enclosure used.

Calves typically nursed around 2 h after birth, with a median duration of 127 min. This follows what is seen in horses, with foals expected to stand by 1 h after birth and nurse by 2 h [51]. Hermes’s study reports a slightly longer time of 3.5 h until successful nursing [42]. The first time to urination was only determined in two of the calves, which occurred at 3.2 h and 6.5 h after birth, respectively. The passage of the meconium was reported in four calves, with a median value of 42.3 h. This is much longer than what is expected in horses, in which the meconium should be passed within 3 h after birth; otherwise, there is the concern for fecal impaction which may require enema treatment. Reports of the first urination or defecation in rhinoceros are rare in the literature. One description of a Sumatran calf reported that the animal did not pass meconium until two weeks of age. The author posited that this was likely physiologic, as other neonates of forest-dwelling species, such as okapi, may not defecate for the first 1–2 months of life [46].

## 5. Conclusions

1. The frequencies of the anterior and posterior presentation in the calves were similar between the GOHR and SWR;
2. Posterior presentation of the calf was associated with a 2.5-times longer phase II of parturition in the GOHR compared to the SWR;
3. The observed frequency of stillbirth was five times higher in the GOHR compared to the SWR;
4. Dystocia and retained placentas were rare compared to horses;
5. Rhinoceros dams will give birth in both lateral recumbency and while standing;
6. Calves are typically standing within an hour after birth and are nursing 1–2 h later; however, the SWR calves stood twice as fast compared to the GOHR calves;
7. The proposed start and end points for the three phases of parturition in rhinoceros are as follows: Phase I begins with two or more signs of impending parturition, such as restlessness and tail flagging/curling, and it ends with the presence of the amniotic sac present at the vulvar lips. Phase II begins with the presence of the amniotic sac at the vulvar lips and ends with the expulsion of the fetus. Phase III begins with the expulsion of the fetus and ends with the passage of the fetal membranes.

## Figures and Tables

**Table 1:** Late-gestation anatomic changes of black rhinoceros (*Diceros bicornis*), greater one-horned rhinoceros (*Rhinoceros unicornis*), and southern white rhinoceros (*Ceratotherium simum simum*) ^1^.

Variable	All Species (n = 47)	Black (*D. bicornis*) (n = 4)	Greater One-Horned (*R. unicornis*) (n = 21)	Southern White (*C. simum simum*) (n = 22)	*p* Value ^2^
Udder development prior to birth (days)					ND
n	5	1	2	2	
Median	21	12	43	12	
Minimum, maximum	2, 60		25, 60	2, 21	
Vulvar edema prior to birth (days)					ND
n	4	1	1	2	
Median	19	10	26	19	
Minimum, maximum	10, 26			17, 21	
Milk leakage prior to birth (days)					ND
n	4	0	3	1	
Median	1.5		1	15	
Minimum, maximum	1, 15		1, 2		

^1^ Data are reported as n, median, minimum, and maximum. ^2^ Statistical comparisons apply to greater one-horned rhinoceros vs. southern white rhinoceros. ND: not determined.

**Table 2:** Signs of impending parturition of black rhinoceros (*Diceros bicornis*), greater one-horned rhinoceros (*Rhinoceros unicornis*), and southern white rhinoceros (*Ceratotherium simum simum*) ^1^.

Variable	All Species (n = 19)	Black (*D. bicornis*) (n = 2)	Greater One-Horned (*R. unicornis*) (n = 7)	Southern White (*C. simum simum*) (n = 10)
Restlessness	13 (68)	2	5	6
Vocalizing	3 (16)	0	3	0
Agitation/aggression	6 (32)	1	3	2
Horn pressing	2 (11)	0	0	2
Tail flagging/curling	8 (42)	0	1	7
Hind-end rubbing/pressing	7 (37)	0	1	6
Kicking hindlimbs	3 (16)	2	0	1
Frequent urination/defecation	4 (21)	1	0	3
Pawing	1 (5)	0	0	1
Anorexia	1 (5)	0	0	1
Isolating behavior	1 (5)	0	0	1
Rolling/abdominal stretching	2 (11)	0	2	0

^1^ Data are presented as observed frequencies (n (%)).

**Table 3:** Gestation lengths and labor phase durations of black rhinoceros (*Diceros bicornis*), greater one-horned rhinoceros (*Rhinoceros unicornis*), and southern white rhinoceros (*Ceratotherium simum simum*) ^1^.

Variable	All Species (n = 47)	Black (*D. bicornis*) (n = 4)	Greater One-Horned (*R. unicornis*) (n = 21)	Southern White (*C. simum simum*) (n = 22)	*p* Value ^2^
Gestation length (days)					<0.01
n	11	1	5	5	
Median	489	454	482	504	
Minimum, maximum	454, 511		467, 489	493, 511	
Phase I time (min)					0.96
n	18	1	8	9	
Median	153	118	145	197	
Minimum, maximum	41, 1770		108, 1770	41, 1440	
Phase II time (min)					0.13
n	21	2	9	10	
Median	28	63	31	20	
Minimum, maximum	5, 200	12, 115	7, 200	5, 46	
Phase III time (min)					0.57
n	15	1	6	8	
Median	205	330	119	219	
Minimum, maximum	30, 343		35, 343	30, 297	

^1^ Data are reported as n, median, minimum, and maximum. ^2^ Statistical comparisons apply to greater one-horned rhinoceros vs. white rhinoceros. ND: not determined.

**Table 4:** Delivery characteristics of black rhinoceros (*Diceros bicornis*), greater one-horned rhinoceros (*Rhinoceros unicornis*), and southern white rhinoceros (*Ceratotherium simum simum*) ^1^.

Variable	All Species (n = 47)	Black (*D. bicornis*) (n = 4)	Greater One-Horned (*R. unicornis*) (n = 21)	Southern White (*C. simum simum*) (n = 22)	*p* Value ^2^
Calf presentation					0.85
Anterior	17 (59)	1 (33)	7 (64)	9 (60)	
Posterior	12 (41)	2 (66)	4 (36)	6 (40)	
Calf position					0.23
Dorsosacral	25 (96)	2 (100)	9 (90)	14 (100)	
Dorsosacral-iliac	1 (4)	0 (0)	1 (10)	0 (0)	
Calf posture					0.42
Hindlimb extended	11 (41)	1 (50)	3 (30)	6 (43)	
Head forelimb extended	16 (59)	1 (50)	7 (70)	8 (57)	
Dystocia					0.09
No	33 (94)	2 (100)	12 (86)	19 (100)	
Yes	2 (6)	0 (0)	2 (14)	0 (0)	
Assisted delivery					1.00
No	37 (100)	3 (100)	14 (100)	20 (100)	
Yes	0 (0)	0 (0)	0 (0)	0 (0)	
Stillbirth					0.07
Yes	6 (13)	0 (0)	5 (24)	1 (5)	
No	40 (87)	4 (100)	16 (76)	21 (95)	
Retained placenta					1.00
No	14 (88)	0 (0)	7 (88)	7 (88)	
Yes	2 (12)	0 (0)	1 (12)	1 (12)	
Dam position during labor					0.24
Lateral recumbency	21 (80)	0 (0)	10 (100)	11 (79)	
Standing	5 (20)	2 (100)	0 (0)	3 (21)	
Calf sex					0.88
Female	12 (37)	0 (0)	7 (41)	5 (39)	
Male	20 (63)	2 (100)	10 (59)	8 (62)	

^1^ Data are presented as observed frequencies (n (%)). ^2^ Statistical comparisons apply to greater one-horned rhinoceros vs. southern white rhinoceros.

**Table 5:** Comparisons of phase I, II, and III (minutes) between calves with anterior and posterior presentation within rhinoceros species ^1^.

Variable	All Species (n = 47)	Black (*D. bicornis*) (n = 4)	Greater One-Horned (*R. unicornis*) (n = 21)	Southern White (*C. simum simum*) (n = 22)
PHASE I				
Anterior				
n	9	0	5	4
Median	135		135	142
Minimum, maximum	95, 360		108, 360	95, 205
Posterior				
n	8	1	3	4
Median	219	118	240	234
Minimum, maximum	41, 1770		130, 1770	41, 1440
*p* value ^2^	0.37	ND	0.39	0.48
PHASE II				
Anterior				
n	11	0	6	5
Median	21		22	12
Minimum, maximum	5, 68		7, 68	5, 29
Posterior				
n	8	1	3	4
Median	39	12	80	31
Minimum, maximum	11, 200		55, 200	11, 46
*p* value ^2^	0.04	ND	0.04	0.11
PHASE III				
Anterior				
n	7	0	4	3
Median	175		119	205
Minimum, maximum	35, 297		35, 291	175, 297
Posterior				
n	4	0	1	3
Median	240		343	233
Minimum, maximum	135, 343			135, 247
*p* value ^2^	0.31	ND	ND	1.00

^1^ Data are reported as n, median, minimum, and maximum. ^2^ Statistical comparisons apply within all species, greater one-horned rhinoceros, or southern white rhinoceros. ND: not determined.

**Table 6:** Duration to calf neonatal milestones of black rhinoceros (*Diceros bicornis*), greater one-horned rhinoceros (*Rhinoceros unicornis*), and southern white rhinoceros (*Ceratotherium simum simum*) ^1^.

Variable	All Species (n = 47)	Black (*D. bicornis*) (n = 4)	Greater One-Horned (*R. unicornis*) (n = 21)	Southern White (*C. simum simum*) (n = 22)	*p* Value ^2^
Calf time to sternal (min)					0.04
n	18	2	6	10	
Median	7	3	17	4	
Minimum, maximum	1, 105	2, 5	7, 105	1, 38	
Calf time to standing (min)					0.02
n	19	2	8	9	
Median	52	54	64	30	
Minimum, maximum	20, 195	53, 56	22, 195	20, 60	
Calf time to nursing (min)					0.29
n	15	2	7	6	
Median	127	203	154	124	
Minimum, maximum	65, 450	117, 290	65, 450	77, 191	
Calf time to urination (min)					ND
n	2		1	1	
Median	291		193	389	
Minimum, maximum	193, 389				
Calf time to passing meconium (min)					ND
n	4	1	1	2	
Median	2535,	4320	2880	1890	
Minimum, maximum	1590, 4320			1590, 2190	

^1^ Data are reported as n, median, minimum, and maximum. ^2^ Statistical comparisons apply to greater one-horned rhinoceros vs. southern white rhinoceros. ND: not determined.

## Data Availability

The data that support the findings of this study are available from the corresponding author upon reasonable request.
